# Pure Orbital Floor Blow-In Fracture: An Extremely Rare Case

**DOI:** 10.7759/cureus.53399

**Published:** 2024-02-01

**Authors:** Kosuke Akiyama, Youhei Ouchi, Atsushi Hosokawa, Motoki Tamai, Hiroshi Hoshikawa

**Affiliations:** 1 Otolaryngology - Head and Neck Surgery, Kagawa University, Miki-cho, JPN; 2 Plastic Surgery, Kagawa University, Miki-cho, JPN

**Keywords:** hess chart, diplopia, orbital floor, orbital bone fracture, blow-in fracture

## Abstract

A 57-year-old man presented with left diplopia on an upward gaze and ophthalmalgia after hitting the left side of his head. CT revealed a fracture on the left side of the orbital floor without orbital rim fractures and the protrusion of a small bone fragment into the orbit. Hess charts indicated markedly limited vertical movement of the left eye. Based on these findings, the patient was diagnosed with a pure orbital floor blow-in fracture (BIF). Symptoms persisted after a two-week monitoring period; therefore, the bone fragment was removed by a transcutaneous surgical approach with the assistance of a navigation system and an endoscope. Symptoms resolved after surgery, and CT and Hess examinations six months after surgery showed a good outcome. A pure BIF is rare, particularly on the orbital floor. Only a few similar case reports have been published to date, and we herein describe the surgical procedures performed and the treatment outcome of our case.

## Introduction

Orbital bone fractures occur in 4-16 % of all facial fractures [1]. The majority of cases present with blow-out fractures (BOFs), which involve bone fragments that have been driven outward, and they are commonly encountered in daily medical practice. These fractures cause functional disability and cosmetic deformity, and patients with obvious complications, such as diplopia or enophthalmos, require surgical repair [2]. In contrast, blow-in fractures (BIFs), in which bone fragments have been inwardly displaced resulting in a decreased orbital volume, are rare [3,4]. In addition to eye movement disorders generally caused by BOFs, serious symptoms, such as eye rupture and superior ophthalmic vein syndrome, may also occur in BIFs [4,5]. BIFs may be further classified as pure or impure depending on whether the orbital rim is involved [5]. Since it is common to have concurrent fractures in other facial bones in BIFs, a pure BIF is rare, and cases occurring on the orbital floor are very rarely encountered [6]. We herein present an extremely rare case of a pure orbital floor BIF. The patient underwent surgery and we provide a detailed description of the surgical procedures performed, treatment progress, and outcome.

## Case presentation

Patient details

**Figure 1:**
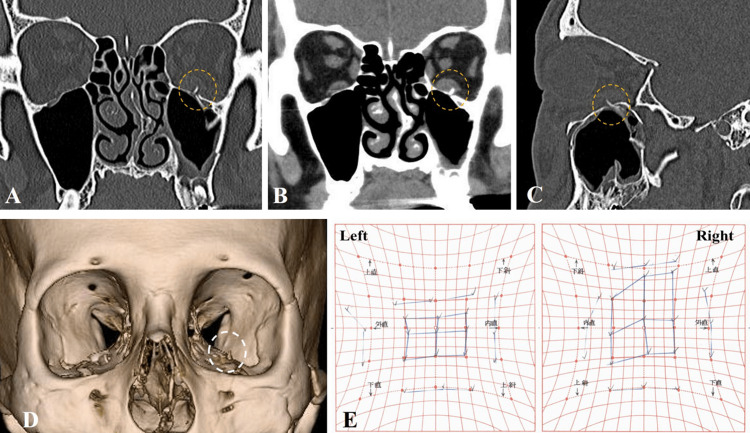
Preoperative CT and Hess charts. Images five days after the injury. Bone window coronal CT (A), soft tissue window coronal CT (B), bone window sagittal CT (C), and 3D-reconstruction CT (D) show a linear fracture of the left orbital floor and the protrusion of a small bone fragment into the orbital space (dot circle). (E) Hess charts showing the significant limitation of left eye movement in the upper gaze.

A 57-year-old man fell while riding his bicycle and hit the left side of his head on the ground. He visited an ophthalmology clinic with diplopia on an upward gaze and ophthalmalgia. The ophthalmologist suspected an orbital bone fracture resulting in an ocular motility disorder and, thus, referred the patient to our hospital for further examination on the fifth day after the injury occurred. Radiographic computed tomography (CT) revealed a small fracture of the left orbital floor and the protrusion of a small bone fragment into the orbit (Figures 1A-1D). Hess charts showed markedly limited vertical eye movement, and the Hess area ratio (%HAR) was 76.6% (Figure 1E). Since symptoms persisted after a two-week monitoring period and re-examinations using CT and Hess charts showed no significant improvement from the initial examination, we decided to proceed with surgical treatment.

Surgical procedures

**Figure 2:**
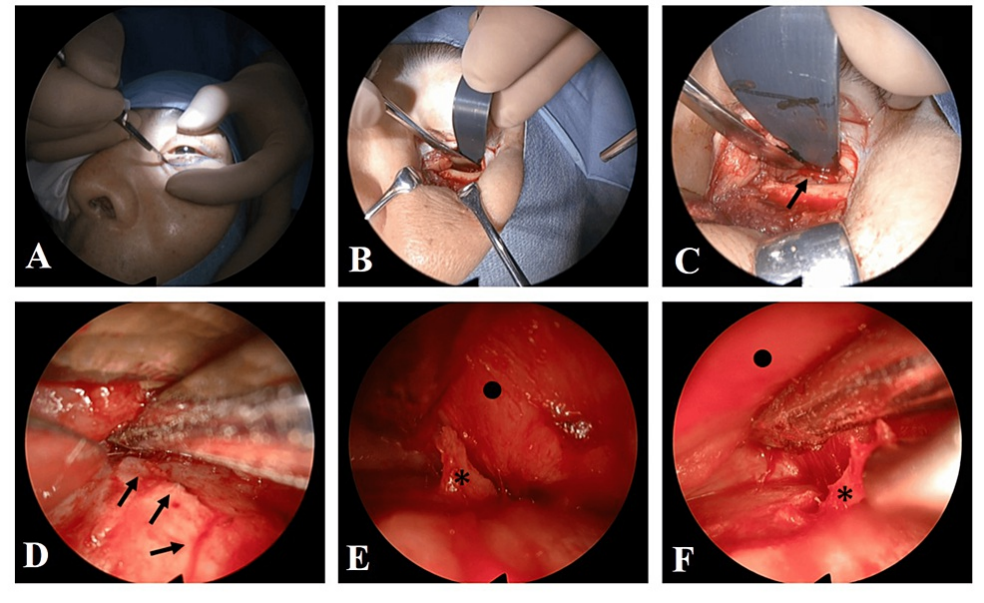
Intraoperative views. (A) Making a subciliary incision. (B) The periosteum was cut and elevated to expose the orbital floor. (C) The fracture line on the orbital floor was confirmed (→). (D) Orbital contents were exfoliated from orbital floor bone under endoscopic assistance. (E) A small piece of bone (*) protruding into the orbital contents was exposed and carefully removed. (F) Removal of the bone fragment.

On the 18th day, surgery was performed by a team of otolaryngologists and plastic surgeons under general anesthesia. A transcutaneous approach to the orbital floor was selected. After making subciliary incisions, the periosteum was incised and elevated and the orbital floor was exposed. The fracture line on the orbital floor extending toward the back was easily confirmed. All subsequent procedures were performed endoscopically by an otolaryngologist while using a navigation system (Stealth Station™ ENT ®, Medtronic Japan, Tokyo, Japan) to ensure proper positioning. A small piece of bone was present at the anteromedial aspect of the inferior orbital fissure and protruded into the orbital contents. The bone fragment was carefully removed from the orbital tissue and the surrounding adhesive tissue was peeled away. A forced duction test at the beginning of surgery showed strong resistance when turning upward; however, this decreased following the removal of the bone fragment (Figure 2 and Video 1).

**Video 1:** Intraoperative view. The surgical procedure for a patient presenting with left orbital floor blow-in fracture.

Postoperative course

**Figure 3:**
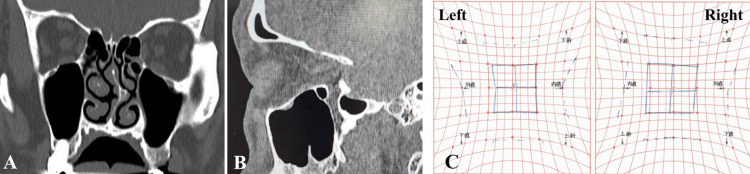
Postoperative CT and Hess charts. (A, B); Coronal and sagittal CT six months after surgery. The bone fragment observed before surgery disappeared and there was almost no bone defect in the left orbital floor. (C); Hess charts six months after surgery showing the good recovery of left eye movement.

Ophthalmalgia disappeared immediately after surgery. Diplopia gradually improved and was nearly completely resolved three months after surgery. Six months after surgery, the patient’s postoperative course has been favorable with no specific complications. Figures 3A-3C show postoperative CT images and Hess charts (%HAR=96.4%).

## Discussion

**Figure 4:**
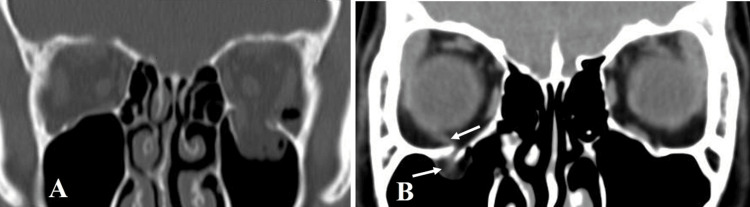
Typical CT images of an orbital floor blow-out fracture. (A) The left orbital floor bone is punched out outwardly and orbital soft tissues fall into the maxillary sinus. Emphysema presents within the left orbit. (B) Linear fracture of the right orbital floor bone with strangulation of the orbital contents, including the inferior rectus muscle (→). Emergency surgery is indicated for such cases.

The BIF and BOF both appear to be similar (Figure 4); however, the BIF is considered to be caused by high-energy blunt damage. The BIF is classified as impure or pure depending on the presence or absence of an orbital rim fracture [6]. A pure BIF is rare among BIF cases, particularly pure orbital floor BIF, with only three cases being reported to date [6-8]. In two of these cases, the clinical course was unclear due to a lack of information from images and on the treatment course, while the other case was reported as a BIF, but appeared to be a BOF based on CT images. Therefore, this case report is one of the few to provide detailed information on the clinical features, treatment procedures, and treatment outcomes of a pure orbital floor BIF.

The underlying mechanisms remain unclear but may include direct external force, indirect external force, and buckling force [9]. The BIF caused by direct external force is most likely to occur at the lateral orbital wall. Indirect external force refers to a sudden increase in pressure in the sinuses or anterior cranial fossa caused by severe trauma, resulting in an inward fracture toward the orbit. Buckling is a sudden change in the shape of a structural component under compression or shear load and is considered to be the most common cause of BIF [6]. In the present case, buckling force was assumed to have been applied to the orbital floor due to a temporal bone bruise, resulting in a fracture in the medial portion of the infraorbital canal, which is the thinnest part.

There is currently no established treatment policy, including the optimal timing for surgery. The BIF is sometimes accompanied by severe symptoms, and early surgery is recommended to reduce intraorbital pressure if there is a risk of eye rupture or optic nerve compression or if symptoms are severe [10]. Although surgical treatment is selected for most cases of BIF, conservative management is sometimes chosen [11]. In the present case, clinical symptoms and laboratory findings were mild, and the bone fragment in the orbit was minute. Therefore, there was no significant increase in intraorbital pressure, and immediate surgery was not considered to be necessary based on BIF and BOF criteria. In cases of the BOF without obvious soft tissue entrapment, it is reasonable to prolong decisions regarding surgery until after an observation period of approximately 1-2 weeks after the injury [12]. Furthermore, no significant differences were noted in postoperative outcomes when surgery was performed within 28 days of the injury [12-14]. These findings were attributed to diplopia being exacerbated in the early period after the injury due to intraorbital emphysema, hematoma, and the swelling of intraorbital soft tissues as well as symptoms spontaneously improving with the attenuation of these conditions. In the present case, subjective symptoms did not improve after a two-week follow-up, and re-testing with the Hess chart only showed a slight improvement. The culprit lesion for symptoms was considered to be the microfracture fragment and, thus, surgery was necessary. Surgical treatment was performed on an elective basis, which followed the usual treatment policy for the BOF [12,15]. There are various approaches to the inferior orbital wall, including transmaxillary sinus, transconjunctival, and percutaneous approaches. There are no clear distinctions for selecting a treatment approach and the one with which the surgeon is accustomed is often selected. In the present case, we used a percutaneous anterior approach, which we considered to be optimal because it was possible to remove fracture fragments with accuracy and minimize bone loss in the orbital floor. Moreover, since the lesion was located within the orbit, an anterior approach was more suitable than a transmaxillary approach. The disadvantage of the anterior approach is that it is challenging to manipulate the retroorbital region [16]. However, by approaching the area posteriorly under an endoscope, we were able to achieve good maneuverability and secure the surgical site. Additionally, the use of a navigation system ensured safety and facilitated the detection of small bone fragments.

## Conclusions

The treatment of BIFs involves healthcare professionals in various departments, such as otolaryngologists, plastic surgeons, ophthalmologists, maxillofacial surgeons, and neurosurgeons, depending on their location and symptoms. The development of an appropriate treatment plan is needed to avoid excessive or unnecessary treatment. Since a BIF is uncommon, a more detailed understanding and further recognition are required.
